# Only two subscales of the Coping Strategies Questionnaire are culturally relevant for people with chronic low back pain in Nigerian Igbo populations: a cross-cultural adaptation and validation study

**DOI:** 10.1186/s41687-021-00367-1

**Published:** 2021-09-08

**Authors:** Chinonso Nwamaka Igwesi-Chidobe, Isaac Olubunmi Sorinola, Emma Louise Godfrey

**Affiliations:** 1 Department of Medical Rehabilitation, Faculty of Health Sciences and Technology, College of Medicine, University of Nigeria (Enugu Campus), Nsukka, Nigeria; 2 Department of Physiotherapy, School of Population Health Sciences, Faculty of Life Sciences and Medicine, King’s College London, London, UK; 3 Department of Psychology, Institute of Psychiatry, Psychology and Neuroscience, King’s College London, London, UK

**Keywords:** Pain coping strategies, Chronic low back pain, Cross-cultural, Nigeria, Africa, Rural

## Abstract

**Background:**

Pain coping strategies are important in the chronicity of low back pain and the associated disability. However, their exact influence is unknown in many African contexts such as rural Nigeria due to lack of outcome instruments with which to measure them. This study aimed to cross-culturally adapt and psychometrically test the Coping Strategies Questionnaire (CSQ) in Igbo populations in Nigeria.

**Methods:**

The CSQ was forward and back translated by clinical and non-clinical translators; evaluated by an expert review committee. The translated measure was piloted amongst twelve rural Nigerian dwellers with chronic low back pain (CLBP) using the think-aloud cognitive interviewing style. Internal consistency (Cronbach’s alpha), test–retest reliability (intra-class correlation coefficient—ICC and Bland–Altman plot), and minimal detectable change were examined amongst 50 people with CLBP in rural and urban Nigerian populations. Construct validity was determined by assessing the correlations between the adapted CSQ and measures of disability, pain intensity, fear avoidance beliefs, and illness perceptions using Spearman’s correlation analyses with 200 adults with CLBP in rural Nigeria. Exploratory factor analyses using Kaiser criterion (eigenvalue) and parallel analysis as methods for determining dimensionality were conducted with the same sample.

**Results:**

Fourteen out of 42 items were routinely adopted in this population including all items of catastrophising subscale, and all but one item of praying and hoping subscale. Catastrophising and praying and hoping subscales had the highest Cronbach’s alpha. All subscales had high ICCs with Bland–Altman plots that showed good agreement. All coping strategies were positively correlated with self-reported disability and pain intensity with catastrophising subscale having the highest values. Seven-factor and three-factor structures were produced with the Kaiser criterion and parallel analysis, with different items from the original CSQ, except for catastrophising.

**Conclusions:**

Catastrophising and praying and hoping may be the relevant coping strategies in this population. More culturally relevant measures of pain coping strategies that include adaptive coping strategies may need to be developed for African contexts such as rural Nigeria.

**Supplementary Information:**

The online version contains supplementary material available at 10.1186/s41687-021-00367-1.

## Background

Pain coping is the effortful response or strategy utilised by an individual to manage the external or internal strains imposed by pain being experienced. Passive pain coping involves withdrawal or relinquishing control of the pain being experienced to an external agent expected to relieve pain. In contrast, active coping requires an individual to initiate instrumental action to address the pain being experienced. Systematic review evidence suggest that passive coping strategies are important contributors to chronic low back pain (CLBP) disability [1]. Passive pain coping strategies have been shown to include depending on others for daily tasks, perceived inability to control pain, hoping for better pain medications from doctors [2]; distraction and praying, helplessness and hopelessness [3] in high income countries.

Distraction and praying/hoping have been shown to be more predictive of pain intensity, whereas ignoring pain sensations and coping self-statements have been more associated with disability [4]. Diverting attention and praying/hoping are associated with greater pain, disability, depression, and pain-related anxiety, less uptime, and worse work status in another study [5]. Praying, hoping, and catastrophising have been associated with more anticipatory anxiety, greater anxiety during painful activity, and reduced range of motion from the onset of pain increase to the point of pain tolerance [6]. Contradictory findings were found in a study showing that increased use of praying and hoping strategies following treatment were significantly related to decreases in reported pain intensity [7]. However, the use of a non-validated pain diary of verbally reported pain intensity, and merging praying/hoping with diverting attention may have influenced findings in the latter. Diverting attention is sometimes useful in CLBP [8]. Passive coping strategies such as focusing on pain, restricting social activities, and depending on pain medication have been consistently associated with poor CLBP outcomes including disability and sick leave [9–12].

Conversely, active pain coping strategies are believed to be adaptive. They have been characterised as staying busy or active, distracting attention from the pain and taking part in physical activity, exercise or physiotherapy, and are associated with neither an increase nor decrease in the risk of developing a new episode of LBP [13] nor did they predict sick leave [9]. The use of coping self-statements such as telling oneself that you can cope with the pain regardless of intensity, was associated with lower skin conductance during anticipation of pain, and greater range of motion [6]. In another study in America, coping self-statements were labelled as denial of pain, and were not associated with positive outcomes [7], similar to findings in urban South Africa [14, 15]. Diverting attention was associated with increased pain intensity, while helplessness was related to depression and functional impairment in the USA based study [7].

Active and passive coping strategies may differ in different contexts due to cultural differences in coping with pain [16]. Hence, the relative importance of different coping strategies may well vary in different cultures and according to the outcome measures included. In addition, temperament traits influence pain experience and behaviour [17]. For instance, maladaptive pain coping strategies can be associated with personality characteristics, in particular, high levels in Harm Avoidance (cautious, fearful, tense, apprehensive, nervous, doubtful, insecure, passive or pessimistic) and low levels in Self-Directedness (immature, weak, fragile, destructive, irresponsible, unreliable, and poorly integrated when not conforming to the direction of a mature leader) [18]. Harm Avoidance is associated with pain-related anxiety including pain-catastrophising, sometimes regarded as a coping strategy, which can be associated with physical inactivity and disuse which can further worsen disability and pain intensity [19]. Moreover, personality characteristics may vary across cultures [20]. Pain medication dependence, searching for permanent cure, and activity pacing were a few of the pain coping strategies highlighted in qualitative studies conducted in rural Nigeria [21, 22]. The influence of pain coping strategies on CLBP disability have not been quantitatively investigated in rural Nigeria possibly due to lack of culturally sensitive measures.

### Aims

There are currently no outcome tools for measuring pain coping strategies in the Igbo Nigerian population. The Coping Strategies Questionnaire (CSQ) is the most widely used self-report measure of pain coping strategies. Therefore, this study aims to:

- Cross-culturally adapt the CSQ into Nigerian Igbo.
- Psychometrically test the CSQ in rural and urban Igbo populations in Nigeria.

## Methods

### Study designs

Translation and cultural adaptation, test–retest measurements, and cross-sectional study of the psychometric properties of the CSQ were performed among Igbo populations with chronic low back pain living in rural and urban settings in Nigeria.

### Ethical issues

Ethical approvals were obtained from King’s College London (Ref: BDM/13/14-99) and University of Nigeria Teaching Hospital (Ref: UNTH/CSA/329/Vol.5). Written permission was obtained from the original developers of the questionnaire. Informed consent was sought and obtained from all the participants involved in this study. Participants were attended to in their homes and workplaces and were not given remuneration for participating in the study.

### Outcome measures

#### Coping Strategies Questionnaire (CSQ)

CSQ was developed to assess cognitive and behavioural coping strategies for dealing with pain [23]. Further validation of the CSQ following initial development by the original developers of the measure produced the 42-item version which was obtained by removing the eighth subscale ‘increasing pain behaviours’. The original authors found that the eight subscale had an unacceptable level of internal consistency and recommended the use of the 42-item version of the CSQ as the standard CSQ [23]. CSQ consists of seven subscales with six items each: (diverting attention [items 3, 9, 12, 26, 27, 38], reinterpreting pain sensation [items 1, 4, 10, 16, 29, 41], catastrophising [items 5, 11, 13, 25, 33, 37], ignoring pain sensations [items 17, 19, 21, 24, 30, 35], praying or hoping [items 14, 15, 18, 22, 28, 36], coping self-statements [items 6, 8, 20, 23, 31, 32] and increased behavioural activities [items 2, 7, 34, 39, 40, 42]). Each item has a numeric rating scale ranging from 0 (never do that) to 6 (always do that). Hence each subscale has a maximum score of 36 and a minimum score of 0. A higher score indicates greater use of a particular coping strategy. Additional two items assess overall effectiveness of pain control and ability to decrease pain. The internal consistencies of the subscales range between 0.71 and 0.85 [23].

#### Eleven-point box scale (BS-11)

The BS-11 is an eleven-point numeric scale for pain intensity [24]. It consists of eleven numbers (0 through 10) surrounded by boxes. Zero represents ‘no pain’ and 10 represents ‘pain as bad as you can imagine’ or ‘worst pain imaginable’. It is easy to comprehend and administer, with high test–retest reliability in both literate and illiterate patients with rheumatoid arthritis (ICC = 0.96 and 0.95, respectively). BS-11 has high correlations (0.86–0.95) with the visual analogue scale (VAS) in patients with rheumatic and other chronic pain conditions; and a reduction of 2 points is clinically significant [24].

#### Igbo Roland Morris Disability Questionnaire (Igbo-RMDQ)

The RMDQ is simple to administer, easily understood, and is most suitable for population-based studies [25]. The Igbo-RMDQ [26], adapted from the original version [27], is a 24-item back specific self-report measure. Each item has possible scores of 0 or 1. A total maximum score of 24 signifies the highest disability and 0 denotes no disability. The Igbo-RMDQ has good face and content validity, construct validity (moderately high correlations [r > 0.6] with performance-based disability and pain intensity), internal consistency (α = 0.84), test–retest reliability (intraclass correlation coefficient = 0.80) [26], and responsiveness (2–3-point change from baseline is considered clinically important) [25].

#### Igbo World Health Organisation Disability Assessment Schedule (Igbo-WHODAS 2.0)

The Igbo-WHODAS 2.0 is a 36-item interviewer administered questionnaire that assesses six domains of disability. These include cognition (understanding and communicating), mobility (getting around), self-care (taking care of oneself), getting along with people (good relationship with people), life activities (maintaining an individual’s household or work/school activities) and participation (participating in society and the impact of the health problem on them and their family). Difficulties encountered are measured within the last 30 days. The measure has good face and content validity, construct validity, internal consistency, test–retest reliability and responsiveness. The complex scoring method considers multiple levels of difficulty for each item. It involves summing recoded item scores in each domain, summing all six domain scores, and converting the total score into a value that range from 0 (no disability) to 100 (maximum disability) [28].

#### Igbo fear avoidance beliefs questionnaire (Igbo-FABQ)

The Igbo-FABQ is a sixteen-item back pain-specific self-report tool that measures the level to which pain is believed to be caused or aggravated by general physical activity (FABQ-PA) and work-related activities (FABQ-W) [29]. The two subscale scores give a total score of 66. Greater scores reflect more fear avoidance beliefs [30]. The physical activity subscale (FABQ-PA) has five items, each with a score ranging from 0 (completely disagree) to 6 (completely agree). Item 1 is a distractor and is not scored. The maximum score for FABQ-PA is 24 and the minimum is 0, with higher scores indicating stronger fear avoidance beliefs related to physical activity. FABQ-W has 11 items, each with a score ranging from 0 (completely disagree) to 6 (completely agree). Items 8, 13, 14, 16 are distractors, and do not contribute to total score. The maximum score for FABQ-W is 42 and minimum score is 0 with higher scores indicating stronger fear avoidance beliefs related to work activities. Igbo-FABQ has good face and content validity, construct validity, internal consistency, test–retest reliability and responsiveness [29]. A change of 13 from baseline is clinically significant [31].

#### Igbo Brief Illness Perceptions Questionnaire (Igbo-BIPQ)

The Igbo-BIPQ is a self-report measure of cognitive and emotional illness perceptions [32] adapted from the original English version [33] with eight items (consequences, timeline, personal control, treatment control, identity, illness concern, coherence and emotional representation), each of which assesses one dimension of illness perceptions. There is an incremental ten-point scale in each item, anchored at 0 and 10 depicting minimal and maximal level of the assessed dimension. The eight items may be combined as one total score, or each item may be assessed separately to give eight dimensions of illness perceptions [33]. Eighty and 0 are the maximum and minimum total scores. A higher score signals a more threatening view of an illness [33]. The ninth item is open and is the causal item. Igbo-BIPQ has good face and content validity, construct validity, internal consistency, test–retest reliability and responsiveness [32].

### Cross-cultural adaptation

#### Participants

A clinical musculoskeletal physiotherapist (bilingual in English and Igbo, native Igbo speaker) who had been practicing in Nigeria for 18 years; and three non-clinical translators (two native English speakers and one native Igbo speaker; all bilingual in English and Igbo) were involved in the cross-cultural adaptation. Two of the non-clinical translators (one native English speaker and one native Igbo speaker) were linguistic experts. Two English experts (health psychologist and academic physiotherapist) in the United Kingdom, and two Igbo experts (clinical psychologist and clinical physiotherapist) in Nigeria made up the expert review committee.

Twelve adults recruited conveniently from a rural population in Enugu State pre-tested/piloted the adapted measure. They were invited to participate in this study via telephone, but data were collected face-to-face following informed consent. Only participants whose CLBP were non-specific (not due to malignancy, spinal fracture, infection, inflammation, or cauda equina syndrome) were recruited.

#### Procedure

**Fig. 1:**
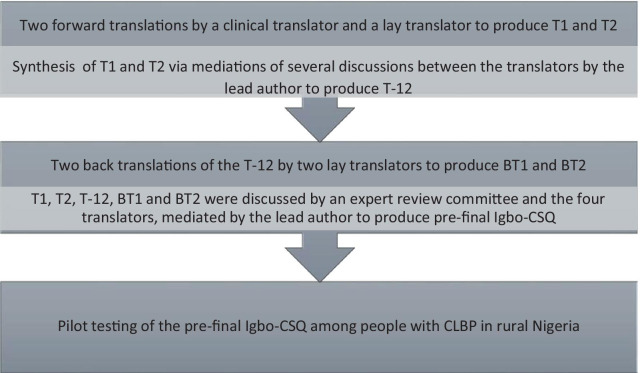
Translation and cultural adaptation stages

The questionnaire was translated and culturally adapted following evidence-based guidelines for a period of one month [34, 35] (Fig. 1).

One bilingual clinical musculoskeletal physiotherapist and one bilingual non-clinical professional translator (native Igbo speakers, bilingual in Igbo and English) forward translated the original CSQ from English to Igbo. Item definitions were provided for the clinical translator to promote understanding of the construct being assessed. Item definitions were not provided for the non-clinical translator to ensure that the translation replicated the lay language used in Igbo culture. T1 and T2 versions of the questionnaire were produced respectively.

A synthesis of T1 and T2 was then performed following discussions between the two forward translators. This was mediated by the first author who is bilingual in English and Igbo to produce a T-12 version. Inconsistencies in the translations were noted by the lead author.

The two back translators back-translated the T-12 CSQ version from Igbo to English. They were both native English speakers from non-clinical backgrounds and blinded to the original measure. One of these back translators was an Igbo/English linguistic expert/professional translator. Hence, BT1 and BT2 back-translated English versions were produced. Back translation validated the translation process ensuring that the translated (T-12) CSQ version was reflecting the construct in the original CSQ.

T1, T2, T-12, BT1 and BT2 versions were subsequently discussed by the expert review committee together with the four translators to produce the pre-final Igbo-CSQ. This committee aimed to achieve semantic, idiomatic, experiential and conceptual equivalence [34]. The expert committee explored Igbo and English words to assess if they meant the same thing, if any item had multiple meanings, and if there were any grammatical difficulties in the translations. The committee helped to formulate alternative Igbo idioms, where English versions were not applicable in the population. The committee also ensured that questionnaire items were experienced similarly in English and Igbo cultures. The committee determined that the words in the items, instructions, and response options had similar conceptual meanings in Igbo and English cultures. They ensured that the Igbo words used were simple and basic.

The pre-final Igbo CSQ was field tested in rural Nigeria among the twelve rural adults. The lead author interviewer-administered the pre-final CSQ using the ‘think-aloud’ cognitive interviewing procedure to assess comprehensibility, acceptability of items and cultural equivalence. Each item was read out by the lead author. Participants were asked to verbalise their thoughts as they tried to answer each question. They were asked if they understood each item, what they understood from each item, the meaning of their chosen response, and if they found any item offensive or irrelevant. They were encouraged to keep verbalising their thoughts while their responses were recorded by the first author. Items that were offensive, irrelevant, or unclear were reviewed by the external review committee together with the translators. This was aimed at ensuring that equivalence was maintained in Nigeria to confirm face and content validity. Technical equivalence was assured via the use of interviewer-administration with all participants.

### Psychometric testing

This took place following the completion of the cross-cultural adaptation phase and lasted for another one month.

#### Sample size

##### Sample for reliability testing

A minimum sample size of 27 will detect an intra-class correlation coefficient of 0.9 at a 95% confidence interval [28]. A convenience sample of 50 people with non-specific CLBP, between the ages of 18 and 69 years, were recruited from rural and urban communities in Enugu State, Nigeria. This sample was used for the estimation of internal consistency (Cronbach’s alpha), test–retest reliability (intra-class correlation coefficient—ICC and Bland–Altman plot), and minimal detectable change.

##### Sample for construct validity investigation

A sample of 194 would give an 80% power to detect a very small correlation coefficient of 0.2 at α level of 0.05. Evaluation of construct validity was done as part of a different study aimed at determining the biopsychosocial factors associated with chronic low back pain disability in rural Nigeria [36]. A representative sample of 200 adults, aged 18 to 69 years, with non-specific CLBP were recruited from rural communities in Enugu State, Nigeria using multistage cluster sampling [36]. A total of ten rural communities from ten rural local government areas were randomly selected. Ten community health workers (CHWs) were recruited and trained to collect data from 20 participants randomly selected from each community, resulting in a total of 200 participants. Informed consent was obtained prior to data collection.

#### Procedure

A training manual was developed, tested and used for training the CHWs for interviewer-administration of the questionnaires. The CHWs were trained on strategies to prevent bias to participants’ responses, and ensure that all questionnaire items were completed. Fidelity checks during data collection ensured that data collection was per protocol. CHWs firstly screened participants using screening questions and a body chart to ascertain that pain was non-specific and in the lower back. Igbo versions of CSQ, BS-11, RMDQ, WHODAS 2.0, FABQ and BIPQ were interviewer-administered with Likert scales presented to participants as ‘flash cards’ as each corresponding item was read out by each CHW.

The Igbo-CSQ was completed at baseline, and repeated 7 to 10 days after, for test–retest reliability investigation amongst the convenience sample of 50 rural and urban participants. The same CHW collected data from each participant on the two occasions.

Igbo versions of CSQ, BS-11, RMDQ, WHODAS 2.0, FABQ and BIPQ were completed at one time-point in a cross-sectional design among the random sample of 200 rural dwellers.

Recruiting different samples enabled a wider applicability of the questionnaire in rural and urban Nigeria, and across literacy levels.

### Data analyses

Data analyses were completed with IBM SPSS version 22 and JASP version 0.14.1. Visual and statistical methods were used to assess data normality.

#### Reliability

Intra-class correlation coefficient (ICC) was used to assess test–retest reliability and evaluated how consistently the Igbo-BIPQ measured illness perceptions over time. A two-way random effects model (with the assumption that measurement errors could arise from either raters or subjects), using an absolute agreement definition between test–retest scores was utilised. Good, very good and excellent ICCs were defined as 0.7, 0.8 and 0.9 respectively [37]. The extent to which all the questionnaire items measure the same construct was investigated using internal consistency (Cronbach’s alpha), and was graded as strong (0.7–1.0), moderate (0.3–0.6) and low/weak (0–0.2) [38]. Visual assessment of the agreement between test–retest measurements were done by plotting mean Igbo-CSQ scores against difference in total Igbo-CSQ scores using Bland–Altman plots. This accounted for the weakness of ICC, which might indicate strong correlations between two measurements with little or no agreement [39].

Standard error of measurement (SEM) and minimal detectable change (MDC) also contributed to reliability investigations. MDC is the smallest change detected by a measure that truly denotes a noticeable change that is not from measurement error. MDC should be sufficiently small to detect minimal clinically important difference [40]. MDC was calculated using the standard error of measurement (SEM) (based on the distribution method), and the reliability of the questionnaire [40]. SEM was estimated using standard deviation (SD) of the sample and the test–retest reliability (R) of the Igbo-CSQ using Eq. (1) below [40]:

1 $${\text{SEM}} = {\text{SD}}\surd \left( {1 - {\text{R}}} \right)$$

Equation (1) *Standard Error of Measurement.*

MDC was estimated with Eq. 2 below:

2 $${\text{MDC}} = {1}.{96}*\surd {2}*{\text{SEM}}$$

Equation (2) *Minimal Detectable Change *where 1.96 = 95% confidence interval of no change; √2 = two measurements [40].

#### Validity

Construct validity is the degree to which an outcome tool measures the construct it was intended to measure [41]. The domain of construct validity assessed was convergent validity using Spearman correlation coefficient (non-parametric data), and was rated as weak (0–0.2), moderate (0.3–0.6), or strong (0.7–1.0). Convergent validity assesses whether two tools that measure constructs that are assumed to be theoretically related, are related indeed. There are no Igbo pain coping tools. Hence, relationships between pain coping strategies and self-reported numeric pain intensity (BS-11), self-reported back pain specific disability (Igbo-RMDQ), self-reported generic disability (Igbo-WHODAS), self-reported fear avoidance beliefs (Igbo-FABQ), and self-reported illness perceptions (Igbo-BIPQ) reported in the literature were used for validity assessment adopting hypotheses set a priori. Regarding the relationships between the CSQ subscales, pain intensity and disability, catastrophising subscale is expected to have at least a moderate correlation with pain intensity measured with the BS-11 and disability measured with the Igbo-RMDQ and Igbo-WHODAS as suggested in people with CLBP [42–44]. Diverting attention, reinterpreting pain sensations, praying or hoping, and increased behavioural activities subscales are expected to have low to moderate correlations with pain intensity measured with the BS-11, and disability measured with the Igbo-RMDQ and Igbo-WHODAS [4–6, 9–12, 45]. Ignoring pain sensations and coping self-statements subscales are not expected to be significantly correlated with pain intensity measured with the BS-11, and disability measured with the Igbo-RMDQ and Igbo-WHODAS [5, 45]. Pain control and pain decrease are expected to be negatively correlated with pain intensity measured with the BS-11, and disability measured with the Igbo-RMDQ and Igbo-WHODAS [6].

Regarding the relationships between the coping strategies and fear avoidance beliefs, negative coping strategies and catastrophising (which may or may not be regarded as a coping strategy) is expected to be positively associated with fear avoidance beliefs [46, 47]. Negative coping strategies include passive coping which are often classified to include praying or hoping, coping self-statements, diverting attention, ignoring pain sensations [3, 5, 6, 9–13] However, the definitions of active, passive, positive, or negative coping strategies appear to differ in different contexts due to cultural differences in coping with pain [16, 48].

Regarding known relationships between coping strategies and illness perceptions, passive coping strategies are known to be stimulated by maladaptive illness perceptions [49]. They are therefore expected to be positively correlated.

Exploratory factor analysis (EFA) was used to determine the number of factors influencing the Igbo-CSQ, that is, its dimensionality [50]. EFA was applied according to Kaiser Meyer Olkin (KMO) and the Bartlett’s test with a minimum eigenvalue for retention set at ⩾1.0 (Kaiser’s rule) [51]. Parallel analysis was included as an additional method for determining the number of factors to be retained in the Igbo-CSQ to compensate for the weakness of the Kaiser criterion which can overestimate or underestimate the number of factors to be retained. In contrast, the parallel analyses shows fewer fluctuations in its accuracy and is more robust [52, 53]. For both methods of determining dimensionality (Parallel analysis and Kaiser criterion), promax (oblique) rotation, which assumes that factors can be related, was done, and factor loadings less than 0.3 were suppressed as recommended; and extraction was done using principal axis factoring as the data had a non-normal distribution [50, 54]. Empirical guidelines are useful, but they are not always correct, and the true number of factors is unknown in reality. Therefore several methods for estimating the number of factors should be utilised and the meanings of findings investigated [52, 53]. The accuracy of empirical guidelines is more likely to be compromised when factors are highly correlated, factor loadings are low, the number of factors is large, and the sample size is small; hence multiple criteria, including relevant theory and previous research, should be used to determine the number of factors to retain [54, 55]. Statistics experts recommend selecting from among a set of competing theoretical explanations the model that best balances the desirable characteristics of parsimony and fit to observed data in terms of interpretability and conceptual sense [54, 55]. Therefore, the number of factors of the adapted measure and their underlying associations were investigated and compared with the original CSQ. Factor pattern coefficients were used for the factor loadings. Scree plot was used for the visual exploration of the retained and excluded factors as recommended [54, 55]. The number of factors and the underlying relationships between Igbo-CSQ items were then compared with the factor structures of the original CSQ to enhance an understanding of population characteristics.

#### Floor and ceiling effects

When a significant number of participants score the maximum or the least score on a measure, ceiling or floor effect occurs. This implies that the two extremes of the scale are not sufficiently differentiated. For this study, 15% or above was regarded as floor or ceiling effect (Lim et al. 2015). This was estimated for each of the seven subscales found in the original CSQ.

## Results

### Participant characteristics

Table 1 describes the sociodemographic characteristics of the participants in the cross-cultural adaptation, test–retest reliability and construct validity samples.

**Table 1:** Demographic characteristics of participants in the three samples

	Age	Gender	Marital status	Main occupation	Religion	Education (years completed)	Literacy	Habitation
Cross-cultural adaptation (pilot/pre-testing) sample; n = 12	45 years (SD 10.36)	Male: 7 (58.3%)	Married: 11 (91.7%) Single: 1 (8.3%)	Non-manual workers: 5 (41.7%) Manual workers: 7 (58.3%)	Pentecostal: 10 (83.3%) Catholic: 2 (16.7%)	10.0 (3.7)	Illiterate: 4 (33.3%) English: 6 (50%) English/Igbo: 2 (16.7%)	Rural
Test–retest reliability sample; n = 50	45.2 years (SD 11.55)	Male: 18 (36.0%)	Married: 37 (74.0%) Single: 8 (16.0%) Widowed: 4 (8.0%) Separated: 1 (2.0%)	Paid Non-manual: 25 (50.0%) Self-employed business/farming: 19 (38.0%) Keeping house/homemaker: 2 (4.0%) Student: 2 (4.0%) Non-paid work/volunteer/charity: 1 (2.0%)		13.3 (7.14)		Urban: 30 (60.0%) Rural: 20 (40.0%)
Construct validity sample; n = 200	48.6 years (SD 12.0)	Male: 112 (44.0%)	Married: 143 (71.5%) Widowed: 31 (15.5%) Single: 22 (11.0%) Cohabiting: 2 (1.0%) Separated: 2 (1.0%)	Self-employed business/farming: 125 (62.5%) Paid Non-manual: 31 (15.5%) Non-paid work/volunteer/charity: 16 (8.0%) Keeping house/homemaker: 13 (6.5%) Student: 7 (3.5%) Unemployed (health reasons): 4 (2.0%) Unemployed (other reasons): 3 (1.5%) Retired: 1 (0.5%)		7.0 (6.4)		Rural: 200 (100%)

### Cross-cultural adaptation findings

For item 1, the Igbo equivalent of ‘I try to forget the pain or behave as if the pain is not in my body…’ was used in place of ‘I try to feel distant from the pain…’ during the synthesis of the forward translations due to lack of an Igbo equivalent for ‘feel distant’. Similarly, in item12, the team used Igbo version of ‘I play some different games in my mind or play mental games…’ in place of ‘I play mental games….’ as the literal translation is an idiomatic Igbo expression that was not understood by everyone especially younger people. For item 29, translators agreed on ‘…is not inside my body’ which echoes the original item because there is no Igbo phrase for ‘…outside of my body’. For item 42, there are no exact Igbo equivalents for ‘active’ and ‘project’ hence the team agreed on ‘I do something that involves moving my body like doing household chores or other works’ to reflect the original item ‘I do something active, like household chores or projects’. Although comprehension of the adapted CSQ was confirmed during verbal pre-testing in rural Nigeria, participants reported not routinely doing the activities in questionnaire items 1, 2, 3, 4, 7, 8, 9, 10, 12, 16, 17, 18, 19, 20, 21, 23, 24, 26, 27, 29, 30, 32, 35, 38, 40, 41, and 42.

### Psychometric properties

**Fig. 2:**
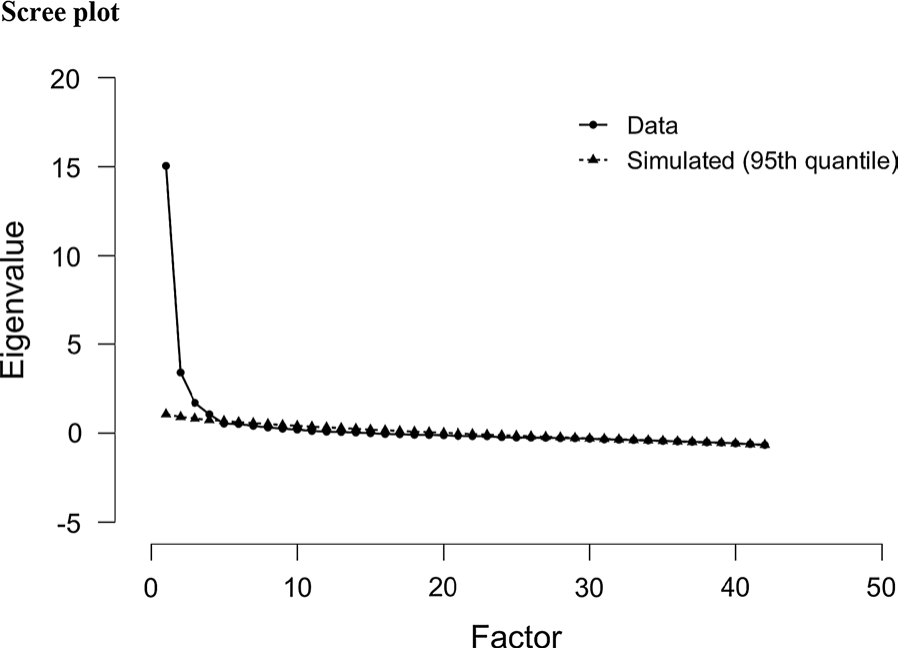
Scree plot of Igbo-CSQ using the parallel analysis for determining dimensionality

No missing data were recorded. Table 2 illustrates the reliability of the adapted CSQ. Bland–Altman plots showed acceptable agreement between test–retest values of the subscales of the Igbo-CSQ as mean differences were close to zero and most points were within the 95% limits of agreement of the mean differences (Additional file 1). Table 3 depicts the construct validity of the adapted CSQ using correlations with measures of disability, pain intensity, fear avoidance beliefs, and illness perceptions. All subscales of the adapted CSQ had moderate correlations with disability (Igbo-RMDQ and the Igbo-WHODAS) and fear avoidance beliefs. Weak to moderate correlations were found between the subscales of the adapted CSQ and illness perceptions (Igbo-BIPQ) except for ignoring sensations, which had no correlations with illness perceptions. The CSQ control and decrease pain items had no significant correlations except for weak positive correlations between CSQ control and disability (Igbo-WHODAS), CSQ control and fear avoidance beliefs, CSQ decrease pain and fear avoidance beliefs. Notably, there was a moderate negative correlation between CSQ control and illness perceptions (Igbo-BIPQ). Table 4 describes the seven-factor structure of the adapted CSQ using the Kaiser criterion for determining dimensionality. 44.64% of the items had factor loadings above 0.5. Factor 1 had main loadings from 4 items of the original reinterpreting pain sensations subscale, 4 items of the original ignoring sensations subscale, 2 items of the original increased behavioural activities subscale, 1 item of the original diverting attention subscale. Factor 2 was loaded mainly by all items of the original praying or hoping subscale, and 4 out of 6 items of the original coping self-statements subscale. Factor 3 had main loadings from 5 out of 6 items of the original diverting attention subscale, and 3 out of 6 items of the original increased behavioural activities subscale. Factor 4 was loaded mainly by all items of the original catastrophising subscale only. Factor 5 was loaded by only 3 items with each item of the original ignoring sensations, coping self-statements and increased behavioural activities subscales. Factor 6 was loaded by 2 items of the original reinterpreting pain sensations subscale, 1 item of the original catastrophising subscale, and one item of the original coping self-statements subscale. Factor 7 was loaded mainly by 1 item of the original ignoring sensations subscale. The catastrophising factor was the only one that retained the structure (100%) of the original measure. Praying and hoping combined with coping self-statements appeared to be one distinct coping strategy as opposed to two strategies in the original measure. Table 5 and Fig. 2 illustrate the three-factor structure of the Igbo-CSQ using the parallel analysis for determining dimensionality. 90.48% of the items had factor loadings above 0.5 but two items (CSQ12 and CSQ31) had no factor loadings (factor pattern coefficients) and were excluded. Factor 1 had loadings from all items of the original reinterpreting pain sensations subscale, increased behavioural activities subscale, ignoring sensations subscale, diverting attention subscale except for one item (CSQ12 which had no factor loading and was excluded), and one item of the original coping self-statements subscale. Factor 2 had loadings from all items of the original praying and hoping subscale and all but two items (4 out of 6 items) of the original coping self-statements (CSQ31 had no factor loading and was hence excluded, and CSQ32 loaded on factor 1). Factor 3 had loadings from all items of the original catastrophising subscale. Once again, the catastrophising factor was the only one that retained the structure (100%) of the original measure, and praying and hoping combined with coping self-statements appeared to be one coping strategy.

**Table 2:** Reliability of Igbo-CSQ

*Igbo-CSQ (diverting attention)* Number of items: 6; Cronbach’s alpha global score: 0.73; ICC (95% CI): 0.89 (0.79, 0.94)							
Cronbach’s alpha if item deleted							
3	9	12	26	27	38		
0.64	0.67	0.71	0.68	0.70	0.71		
SEM: 2.43 MDC: 6.73							
*Igbo-CSQ (reinterpreting pain sensation)* Number of items: 6; Cronbach’s alpha global score: 0.81; ICC (95% CI): 0.93 (0.88, 0.96)							
Cronbach’s alpha If Item Deleted							
1	4	10	16	29	41		
0.80	0.81	0.76	0.79	0.74	0.76		
SEM: 3.58 MDC: 9.92							
*Igbo-CSQ (catastrophising)* Number of items: 6; Cronbach’s alpha global score: 0.85; ICC (95% CI): 0.77 (0.60, 0.87)							
Cronbach’s alpha If Item Deleted							
5	11	13	25	33	37		
0.81	0.83	0.82	0.85	0.80	0.82		
SEM: 2.51 MDC: 6.96							
*Igbo-CSQ (ignoring pain sensations)* Number of items: 6; Cronbach’s alpha global score: 0.66; ICC (95% CI): 0.80 (0.64, 0.89)							
Cronbach’s alpha if item deleted							
17	19	21	24	30	35		
0.69	0.61	0.60	0.54	0.66	0.55		
SEM: 2.96 MDC: 8.20							
*Igbo-CSQ (praying or hoping)* Number of items: 6; Cronbach’s alpha global score: 0.86; ICC (95% CI): 0.90 (0.82, 0.94)							
Cronbach’s alpha if item deleted							
14	15	18	22	28	36		
0.83	0.82	0.86	0.84	0.83	0.86		
SEM: 2.09 MDC: 5.79							
*Igbo-CSQ (coping self-statements)* Number of items: 6; Cronbach’s alpha global score: 0.79; ICC (95% CI): 0.91 (0.84, 0.95)							
Cronbach’s alpha If Item Deleted							
6	8	20	23	31	32		
0.76	0.72	0.79	0.76	0.78	0.74		
SEM: 2.18 MDC: 6.04							
*Igbo-CSQ (increased behavioural activities)* Number of items: 6; Cronbach’s alpha global score: 0.77; ICC (95% CI): 0.91 (0.84, 0.95)							
Cronbach’s alpha If Item Deleted							
2	7	34	39	40	42		
0.76	0.78	0.75	0.67	0.70	0.71		
SEM: 2.52 MDC: 6.98

**Table 3:** Spearman’s correlation between Igbo-CSQ subscales, and self-reported back pain specific disability (Igbo-RMDQ), self-reported generic disability (Igbo-WHODAS), self-reported numeric pain intensity (BS-11), self-reported fear avoidance beliefs (Igbo-FABQ), and self-reported illness perceptions (Igbo-BIPQ)

	Igbo-CSQ (Diverting attention)	Igbo-CSQ (reinterpreting pain sensation)	Igbo-CSQ (catastrophising)	Igbo-CSQ (ignoring sensations)	Igbo-CSQ (praying or hoping)	Igbo-CSQ (coping self-statements)	Igbo-CSQ (increased behavioural activities)	Igbo-CSQ (control)	Igbo-CSQ (decrease pain)
Igbo-RMDQ	0.554**	0.302**	0.614**	0.272**	0.410**	0.333**	0.441**	− 0.027	− 0.030
Igbo-WHODAS (total)	0.391**	0.459**	0.589**	0.371**	0.265**	0.237**	0.324**	0.169*	0.126
BS-11	0.263**	0.255**	0.469**	0.171*	0.292**	0.281**	0.217**	− 0.108	− 0.037
Igbo-FABQ total	0.643**	0.552**	0.492**	0.448**	0.475**	0.451**	0.565**	0.193**	0.208**
Igbo-BIPQ (total)	0.323**	0.193**	0.287**	0.004	0.255**	0.312**	0.309**	− 0.343**	− 0.007

^**^p < 0.01; *p < 0.05

**Table 4:** Exploratory factor analysis of the Igbo-CSQ using the Kaiser criterion to determine dimensionality

	1	2	3	4	5	6	7
CSQ16	0.802						
CSQ21	0.760						
CSQ17	0.736		− 0.364				
CSQ29	0.713						
CSQ1	0.667						
CSQ24	0.564						
CSQ35	0.549						
CSQ41	0.515						
CSQ27	0.480		0.332				
CSQ7	0.450						
CSQ39	0.326						
CSQ36		0.979					
CSQ15		0.964					
CSQ28		0.928					
CSQ22		0.736					
CSQ8		0.620					
CSQ20		0.553					
CSQ14		0.525				0.361	
CSQ6		0.489					
CSQ23		0.486					0.481
CSQ18	0.313	0.393					
CSQ12			0.984				
CSQ38			0.652				
CSQ3			0.595				
CSQ26	0.309		0.561				
CSQ2	0.330		0.530				
CSQ34			0.481				0.317
CSQ9			0.473				
CSQ40			0.467				
CSQ33				0.790			
CSQ5				0.766			
CSQ37				0.743			
CSQ13			0.328	0.633			
CSQ11			0.423	0.488			
CSQ30					0.728		
CSQ32					0.482		
CSQ42			0.316		0.381		
CSQ4	0.339					0.464	
CSQ25				0.390		0.402	
CSQ31		0.300				0.362	
CSQ10						0.346	
CSQ19							0.339
KMO = 0.93							
χ^2^ = 5499.07*** Proportion of explained variance of factor	0.136	0.146	0.115	0.074	0.038	0.041	0.031

Only factor loadings above 0.3 are shown; KMO = Kaiser–Meyer–Olkin measure of sampling adequacy; χ^2^ = Bartlett’s test of sphericity tested with chi-square ***p < 0.001; Extraction Method: Principal Axis Factoring; Rotation Method: Promax with Kaiser Normalization; Rotation converged in 7 iterations

**Table 5:** Exploratory factor analysis of the Igbo-CSQ using the parallel analysis to determine dimensionality

	1	2	3
CSQ1	0.814		
CSQ2	0.641		
CSQ3	0.720		
CSQ4	0.555		
CSQ7	0.589		
CSQ9	0.560		
CSQ10	0.599		
CSQ16	0.745		
CSQ17	0.514		
CSQ19	0.439		
CSQ21	0.669		
CSQ24	0.727		
CSQ26	0.735		
CSQ27	0.622		
CSQ29	0.724		
CSQ30	0.601		
CSQ32	0.503		
CSQ34	0.626		
CSQ35	0.594		
CSQ38	0.633		
CSQ39	0.671		
CSQ40	0.425		
CSQ41	0.763		
CSQ42	0.537		
CSQ6		0.530	
CSQ8		0.647	
CSQ14		0.578	
CSQ15		1.032	
CSQ18		0.423	
CSQ20		0.608	
CSQ22		0.781	
CSQ23		0.539	
CSQ28		0.967	
CSQ36		1.019	
CSQ5			0.794
CSQ11			0.657
CSQ13			0.729
CSQ25			0.485
CSQ33			0.789
CSQ37			0.690
χ^2^ = 1309.099*** Proportion of explained variance of factor	0.259	0.152	0.086

Excluded CSQ items: CSQ12 and CSQ 31

***p < 0.001

### Findings from investigating floor and ceiling effects

11 (5.5%) and 0 (0.0%) people scored 0 and 36 respectively on the original reinterpreting pain sensation subscale. 7 (3.5%) and 0 (0.0%) people scored 0 and 36 respectively on the original catastrophising subscale. 0 (0.0%) and 1 (0.5%) people scored 0 and 36 respectively on the original diverting attention subscale. 1 (0.5%) and 0 (0.0%) people scored 0 and 36 respectively on the original ignoring sensations subscale. 0 (0.0%) and 8 (4.0%) people scored 0 and 36 respectively on the original praying or hoping subscale.

## Discussion

Although translation of the CSQ (Additional file 2) was without complications, and comprehensibility was confirmed among these rural Nigerian dwellers with CLBP, the pilot sample of twelve people reported not adopting most of the activities listed in the questionnaire items in response to pain. Of the 42 items in the questionnaire, only 14 items (5, 6, 11, 13, 14, 15, 22, 25, 28, 33, 34, 36, 37, 39) were reported as commonly adopted in the pilot sample of twelve people. These included all items of the catastrophising subscale, and all but one item of the praying and hoping subscale. In the only praying and hoping item 18 not commonly adopted, ‘I try to think years ahead, what everything will be like after I’ve gotten rid of the pain’, participants wished to be rid of their pain, but did not tend to imagine what a future without their pain would be like. One item from the coping self-statements subscale and two items from the increased behavioural activities subscale were also common in this population. Item 6 of the coping self-statements subscale ‘I tell myself to be brave and carry on despite the pain’, found to be common in the pilot sample of twelve people, has been shown to be adaptive or maladaptive [21]. Those who carried on and increased their activity level too much in an attempt to ‘fight back against their pain’ might report more disability. In contrast, those who carried on with daily activity but paced their activity level reported less disability [21]. Some participants in the pilot sample of twelve people leave their house and perform some activities in response to pain, but these activities do not typically involve going to the movies or shopping as stated in item 2 of the increased behavioural activities subscale. Similarly, although participants sometimes do something they enjoy in response to pain, this was not watching TV or listening to music as stated in item 40 of the increased behavioural activities subscale. Not all the other items were never adopted in this population. Some uncommon items were activities that although are performed in this population, were not usually in response to pain. For instance, although the participants in the pilot sample of twelve people might sometimes do something active like household chores or projects (item 42 of the increased behavioural activities subscale), it was not usually in response to having pain. Previous qualitative studies in this population suggests that this behaviour was often in response to positive beliefs such as not regarding CLBP as an illness which facilitates a relinquishment of the sick role that enabled active behavioural adaptation such as pacing activities of daily living [21]. Hence, catastrophising and praying and hoping appeared to be the only consistently adopted coping strategies in this population.

Of important note is the possible influence of culture on personality trait which can both influence pain perception and expression including pain coping strategies [17–20]. People of African ancestry are said to report higher levels of pain unpleasantness, emotional response to pain and pain behavior, in response to similar levels of pain intensity than other ethnic groups [56–58]. However, other studies have reported little influence of ethnicity on pain experience, after controlling for pain duration, economic, educational and social factors [59, 60]. The Africans in these studies were African Americans and so may not represent Africans in Africa. The possible influence of acculturation and adjustment, with possible differential adverse influence on mental health were not considered in these studies. The personality traits of Nigerian Igbos living with chronic low in Nigeria and their possible influence on pain coping strategies need to be investigated in future studies. A previous qualitative study in rural Nigeria suggests some cultural explanations for persistent back pain [22]. For instance, Nigerian Igbos were believed to have low pain tolerance, which when combined with their perceived inordinately high ambitions, were believed to drive behaviour such as constant working and lack of rest, which maintained pain persistence [22].

Praying and hoping (0.86) and catastrophising (0.85) had the highest Cronbach’s alpha, suggesting that these coping strategies may be more consistent in this population than ignoring pain sensations (0.66) with the least Cronbach’s alpha. This concurs with findings from the cross-cultural adaptation and previous qualitative findings in this population [21]. In contrast, ignoring pain sensations may be more popular than praying and hoping in western settings [61, 62]. All subscales of the adapted CSQ had high ICCs ranging between 0.77 and 0.91 with Bland–Altman plots that showed good agreement.

None of the coping strategies of the adapted CSQ subscales appeared adaptive in this population as they were all positively correlated with self-reported disability and pain intensity. This contradicts findings in western culture where diverting attention and increasing physical activity, ignoring pain sensations, and coping self-statements can be adaptive [5, 8, 45]. Notably, the catastrophising subscale had the strongest positive correlation with pain intensity, and self-reported disability, again suggesting its consistency and significant role in this population. Diverting attention had the strongest correlations with fear avoidance beliefs and illness perceptions suggesting that adopting this coping strategy might be related to higher fear avoidance beliefs and greater threatening view of CLBP. One of the two pain self-efficacy items (pain decrease) did not have any significant negative correlation with any of the outcomes. However, the other pain self-efficacy item (pain control) had a significant negative correlation with illness perceptions. This suggests that there is perception of adequate control of CLBP when CLBP is not viewed as a threatening illness. Previous research showed that illness perceptions and fear avoidance beliefs were the most important predictors of both self-reported and performance-based disability in this population [36]. These associations must however be interpreted with caution considering that most of these subscales (apart from catastrophising and praying and hoping subscales) may lack relevance in this population.

Using the Kaiser criterion to determine dimensionality, a seven-factor solution of the adapted CSQ was produced like the original measure [23]; but items in the factors were different. In contrast, a three-factor solution was produced using the parallel analysis to determine dimensionality. Catastrophising subscale was the only factor reproduced as in the original measure in both methods of determining dimensionality, again suggesting that this is a consistent strategy in this population. However, the exact definition of catastrophising in CLBP is conflicting. It is regarded as a cognitive coping strategy [23, 63–65], or as part of the fear avoidance model [46, 66, 67]. Other authors believe the construct is indistinguishable from negative mood, beliefs, adjustment or contextual pain factors [6, 68–70]. The concepts of emotional distress and pain coping strategies need to be clarified in future studies in this population. Furthermore, praying/hoping and coping self-statements appeared to be one distinct strategy in this population utilising both methods of determining dimensionality. The remaining four coping strategies in the Kaiser criterion, and the remaining one coping strategy in the parallel analysis, did not appear to be defined or consistent in this population. Despite these findings, there were no floor or ceiling effects in any of the original subscales in the adapted Igbo-CSQ. New pain coping strategies measures may need to be developed to reflect how people coped with CLBP in rural African contexts such as rural Nigeria. Patient generated outcomes may also prove useful in measuring coping strategies in these contexts by allowing participants to list all the ways they managed their CLBP which can then be analysed categorically. It is important to identify adaptive coping strategies that may be useful in reducing pain and disability in this population which can then be the focus of complex behaviour change interventions.

### Strengths and limitations

This study enabled the identification of the relevant pain coping strategies in Nigerian Igbo populations, particularly those in rural Nigeria, from the commonly used CSQ. Pilot/field testing of the Igbo-CSQ among participants living with CLBP in rural Nigeria by interviewer-administration using the ‘think-aloud’ cognitive interviewing procedure which allowed participants to identify the relevant and non-relevant coping strategies confirmed face and content validity to an extent. The identified pertinent strategies in the questionnaire can be interviewer-administered, and will have great utility, especially among illiterate rural dwellers with chronic pain, who are often neglected despite being highly vulnerable. Other strengths of the study include acceptable reliability and construct validity characteristics of the relevant coping strategies. Construct validity is supported by the use of multiple measures including that of disability, pain intensity, fear avoidance beliefs, and illness perceptions. The relevant subscales of the Igbo-CSQ can be used to validate new measures of coping strategies in this population. The use of EFA was warranted in this study as the Igbo-CSQ was just adapted for use for the first time in this population. EFA allowed an exploration of the underlying structure of this measure in this new population which can then be tested using confirmatory factor analysis. Specifically, confirmatory factor analysis of the Igbo-CSQ can be conducted in this population in future studies to determine the model fit indices for the observed EFA structure of the Igbo-CSQ found in this study, as well as the model fit indices for the factor structures found in the original CSQ measure and other factor structures of the CSQ reported in the literature. Findings can then confirm the subscales and the structures of the Igbo-CSQ most suitable for this population.

A limitation of this study is the lack of statistical investigation of item redundancy. The lack of statistical and systematic examination of item redundancy could have distorted the content validity.

## Conclusions

This study aimed to cross-culturally adapt and test the CSQ in rural and urban Nigerian populations. This study found that catastrophising and praying and hoping may be the only relevant subscales of the CSQ in Nigeria, and are both maladaptive coping strategies. More culturally relevant measures of pain coping strategies that include adaptive coping strategies may need to be developed for Africa, particularly rural African contexts such as rural Nigeria.

## Supplementary Information

**Additional file 1: Figs. S1–S7**. Bland–Altman test–retest agreement.

**Additional file 2**. Coping strategies questionnaire (original and adaptation).

## Data Availability

Data is available on request due to ethical restrictions imposed by Biomedical and Health Sciences, Dentistry, Medicine and Natural and Mathematical Sciences Research Ethics Subcommittees (BDM RESC) Kings College London. Requests for data access may be made to BDM RESC Kings College London through email bdm@kcl.ac.uk.

## Abbreviations

CSQ: Coping Strategies Questionnaire
LBP: Low back pain
CLBP: Chronic low back pain
ICC: Intra-class correlation coefficient
SEM: Standard error of measurement
MDC: Minimal detectable change
BS-11: Eleven-point box scale
Igbo-RMDQ: Igbo Roland Morris Disability Questionnaire
Igbo-WHODAS: Igbo World Health Organisation Disability Assessment Schedule
Igbo-FABQ: Igbo fear avoidance beliefs questionnaire
Igbo-BIPQ: Igbo Brief Illness Perceptions Questionnaire
CHWs: Community health workers
EFA: Exploratory factor analysis
