# Supplementary material for: Only two subscales of the Coping Strategies Questionnaire are culturally relevant for people with chronic low back pain in Nigerian Igbo populations: a cross-cultural adaptation and validation study
Source: J Patient Rep Outcomes. 2021 Sep 8;5:85. doi: 10.1186/s41687-021-00367-1 (PMC8426442; doi:10.1186/s41687-021-00367-1)
Supplement: Supplementary file 1 — Additional file 1: Figs. S1–S7. Bland–Altman test–retest agreement. [file 41687_2021_367_MOESM1_ESM.docx]

# **Supplemental file 1**


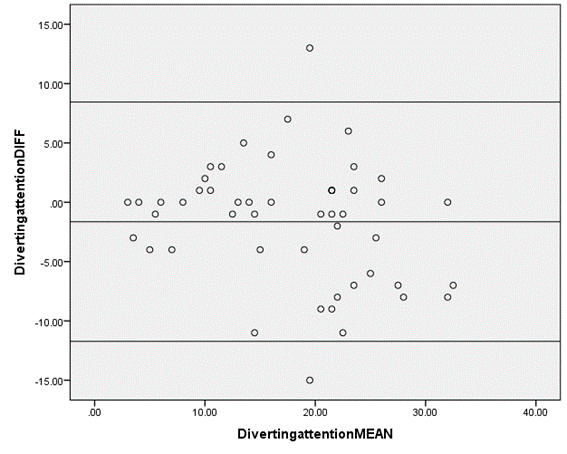


(-1.96 SD): -11.73

-1.64 (-3.10, -0.18) SD: 5.15

(+1.96 SD): 8.45

**Supplemental figure 1:** Bland-Altman plot for test-retest agreement of Igbo-CSQ (diverting attention)


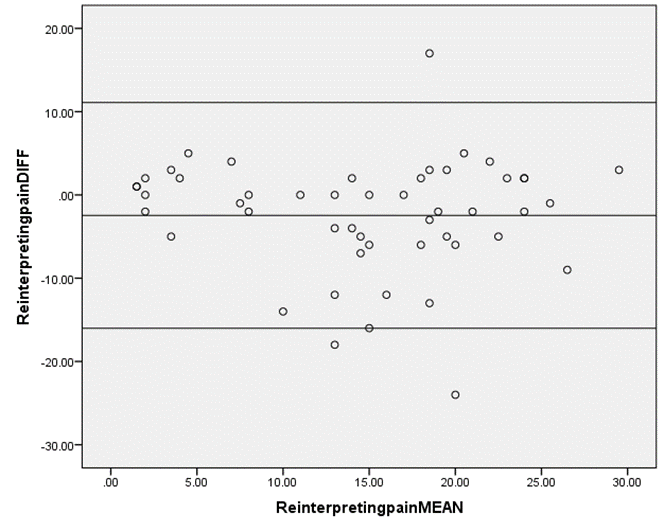


(+1.96 SD): 11.10

(-1.96 SD): -16.02

-2.46 (-4.43, -0.49) SD: 6.92

**Supplemental figure 2:** Bland-Altman plot for test-retest agreement of Igbo-CSQ (reinterpreting pain sensation)


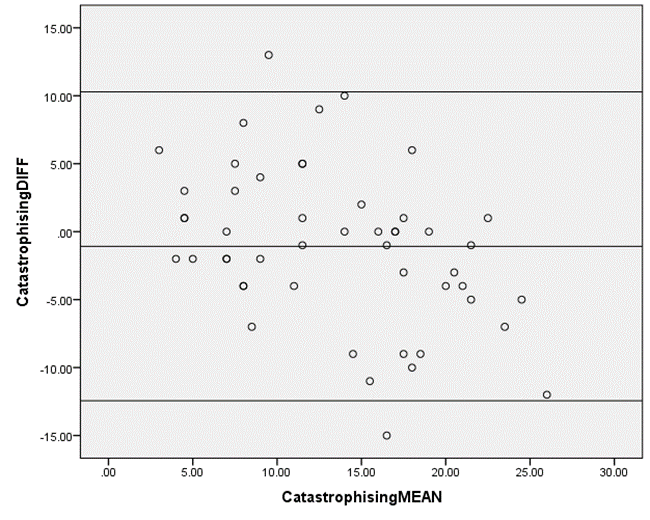


(+1.96 SD): 10.29

(-1.96 SD): -12.45

-1.08 (-2.73, 0.57); SD: 5.80

**Supplemental figure 3:** Bland-Altman plot for test-retest agreement of Igbo-CSQ (catastrophising)


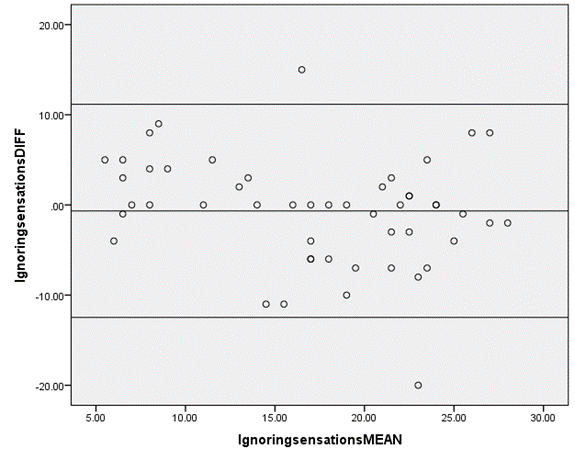


(+1.96 SD): 11.16

(-1.96 SD): -12.48

-0.66 (-2.37, 1.05); SD: 6.03

**Supplemental figure 4:** Bland-Altman plot for test-retest agreement of Igbo-CSQ (ignoring pain sensations)


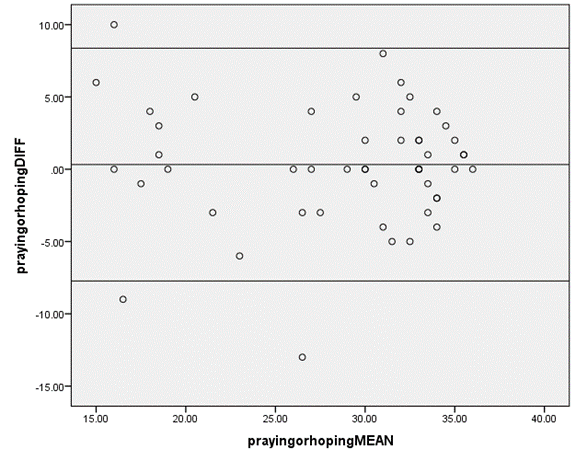


(+1.96 SD): 8.38

(-1.96 SD): -7.74

0.32 (-0.85, 1.49); SD: 4.11

**Supplemental figure 5:** Bland-Altman plot for test-retest agreement of Igbo-CSQ (praying or hoping)


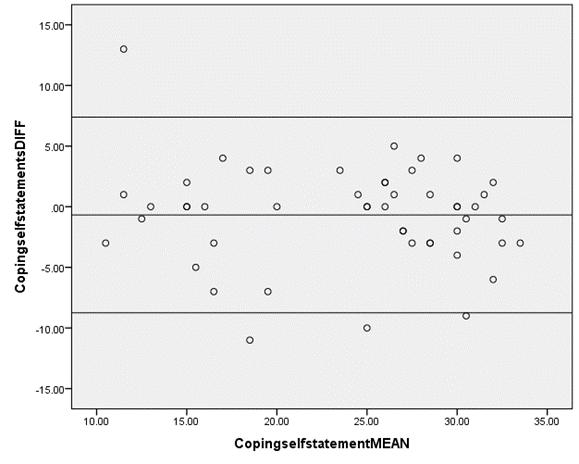


(-1.96 SD): -8.76

-0.68 (-1.85, 0.49); SD: 4.12

(+1.96 SD): 7.40

**Supplemental figure 6:** Bland-Altman plot for test-retest agreement of Igbo-CSQ (coping self-statements)


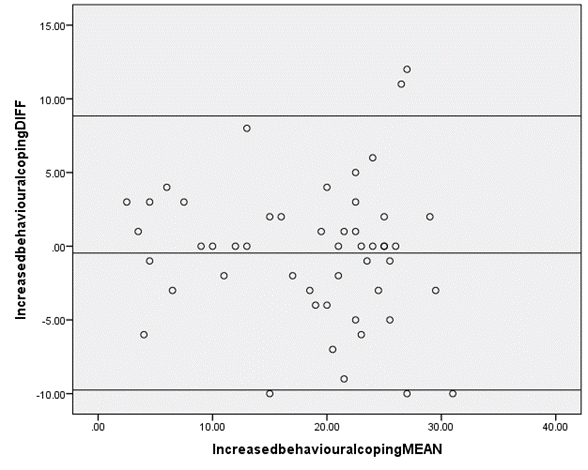


(-1.96 SD): -9.75

(+1.96 SD): 8.83

-0.46 (-1.81, 0.89); SD: 4.74

**Supplemental figure 7:** Bland-Altman plot for test-retest agreement of Igbo-CSQ (increased behavioural activities)
