# Supplementary material for: Only two subscales of the Coping Strategies Questionnaire are culturally relevant for people with chronic low back pain in Nigerian Igbo populations: a cross-cultural adaptation and validation study
Source: J Patient Rep Outcomes. 2021 Sep 8;5:85. doi: 10.1186/s41687-021-00367-1 (PMC8426442; doi:10.1186/s41687-021-00367-1)
Supplement: Supplementary file 2 — Additional file 2. Coping strategies questionnaire (original and adaptation). [file 41687_2021_367_MOESM2_ESM.docx]

# **Supplemental file 2: Original and Igbo pain coping strategies questionnaire**

**ORIGINAL PAIN COPING STRATEGIES QUESTIONNAIRE**

Instructions: Individuals who experience pain have developed a number of ways to cope or deal with their pain. These include saying things to themselves when they experience pain, engaging in different activities. Below is a list of things that patients have reported doing when they feel pain. For each activity, please indicate, using the scale below, how much you engage in that activity when you feel pain, where 0 indicates you NEVER do that when you are experiencing pain, a 3 indicates you SOMETIMES do that when you are experiencing pain, and a 6 indicates you ALWAYS do that when you are experiencing pain. Remember, you can use any point along the scale.

*DA = Diverting Attention; RS = Reinterpreting Pain Sensations; CAT = Catastrophizing; IS = Ignoring Pain Sensations; PH = Praying and Hoping; CSS = Coping Self-Statements; IPB = Increasing Pain Behaviors; IBA = Increased Behavioral Activities; DIS = Distraction.*

NEVER SOMETIMES ALWAYS

0 1 2 3 4 5 6

When I feel pain...

1. I try to feel distant from the pain, almost as if the pain was in someone else’s body (RS)

2. I leave the house and do something, such as going to the movies or shopping (IBA)

3. I try to think of something pleasant (DA)

4. I don’t think of it as pain but rather as a dull or warm feeling (RS)

5. It’s terrible and I feel it’s never going to get any better (CAT)

6. I tell myself to be brave and carry on despite the pain (CSS)

7. I read (IBA)

8. I tell myself I can overcome the pain (CSS)

9. I count numbers in my head or run a song through my mind (DA)

10. I just think of it as some other sensation such as numbness (RS)

11. It’s awful and I feel it overwhelms me (CAT)

12. I play mental games with myself to keep my mind off the pain (DA)

13. I feel my life isn’t worth living (CAT)

14. I know someday someone will be here to help me and it will go away for awhile (PH)

15. I pray to God it won’t last long (PH)

16. I try not to think of it as my body, but rather as something separate from me (RS)

17. I don’t think about the pain (IS)

18. I try to think years ahead, what everything will be like after I’ve gotten rid of the pain (PH)

19. I tell myself it doesn’t hurt (IS)

20. I tell myself I can’t let the pain stand in the way of what I have to do (CSS)

21. I don’t pay any attention to it (IS)

22. I have faith in doctors that someday there will be a cure for my pain (PH)

23. No matter how bad it gets, I know I can handle it (CSS)

24. I pretend it’s not there (IS)

25. I worry all the time about whether it will end (CAT)

26. I replay in my mind pleasant experiences in the past (DA)

27. I think of people I enjoy doing things with (DA)

28. I pray for the pain to stop (PH)

29. I imagine that the pain is outside of my body (RS)

30. I just go on as if nothing happened (IS)

31. I see it as a challenge and don’t let it bother me (CSS)

32. Although it hurts, I just keep on going (CSS)

33. I feel I can’t stand it anymore (CAT)

34. I try to be around other people (IBA)

35. I ignore it (IS)

36. I rely on my faith in God (PH)

37. I feel like I can’t go on (CAT)

38. I think of things I enjoy doing (DA)

39. I do anything to get my mind off the pain (IBA)

40. I do something I enjoy, such as watching TV or listening to music (IBA)

41. I pretend it’s not a part of me (RS)

42. I do something active, like household chores or projects (IBA)

**Based on all the things you do to cope or deal with your pain, on an average day, how much control do you feel you have over it? Please circle the appropriate number. Remember, you can circle any number along the scale.**

0 1 2 3 4 5 6

No control Some control

Complete control

**Based on all the things you do to cope or deal with your pain, on an average day, how much are you able to decrease it? Please circle the appropriate number. Remember, you can circle any number along the scale.**

0 1 2 3 4 5 6

Can’t Can decrease it Can decrease it

Decrease somewhat completely

it at all

**IGBO PAIN COPING STRATEGIES QUESTIONNAIRE**

Ndi mmadu ndi na enwe ahu mgbu achoputala uzo ole na ole ha na eji na-anagide ihe mgbu ha. Nke a gunyere I na-agwa onwe ha okwu nkasiobi mgbe o bula ha na enwe ahu mgbu, isonyere na ufodu emume di iche iche. Ihe na-esota ugbu a bu usoro ndi n’enwe ahu mgbu kowaputara ha na eme mgbe ha na enwe ahu mgbu. Maka ihe omume nke o bula, biko were ihe skelu a (ihe a akara aka) gosi ihe ndi a, etu I na-esi etinye onwe gi na ihe omume ahu mgbe o bula I na enwe ahu mgbu, were ihe dika EFU/ONWEGHI MGBE gosi na INAGHI eme ihe ahu ma oli mgbe o bula I na-enwe ahu mgbu, were akara nke ATO gosi na I na eme ihe ahu MGBE UFODU I na enwe ahu mgbu, were kwa akara nke ISII gosi na I na eme ihe ahu MGBE NILE I na enwe ahu mgbu. Chetakwa na i nwekwara ike I were akara o bula n’ime ihe otutu skelu a (ihe a akara aka) kowaa ya.

EFU/ONWEGHI MGBE MGBE UFODU MGBE NILE

0 1 2 3 4 5 6

Mgbe m na enwe ahu mgbu...

1. Ana m agbali ka m chefue ya ma o bu mee ka o dika na obughi na ahu m ka ahu mgbu ahu di site na ibu na echiche na obu na ahu onye ozo.
2. Ana m esi n’ulo ebe m no puo ga mee ihe dika I ga ahia ma o bu I ga lee ihe nkiri onyo onyooo
3. Ana m agbali chee echiche ihe na enye obi uto.
4. Anaghi m eche ya na o bu ahu mgbu, kama a na m ewere ya na o ihe na emetuta m na ahu ma o bu ihe na ekpo oku.
5. Okacha m njo ma dikwa m ka ogaghi adikwa mma
6. Ana m agwa onwe m, ka m nwee mmuo siri ike ma jisie ike na-agbali n’agbanyeghi ahu mgbu ahu
7. A na m agu akwukwo
8. A na m agwa onwe m na m ga-emeri ihe mgbu a.
9. A na m agu onuogugu n’isi m ma o bu na ekwe ukwe na obi m
10. A na m ewe ya dika ihe ozo na eme m, ihe dika ihe mmekpa ahu ozo dika otita ngwere
11. bu ihe jogburu onwe ya, dikwa m ka o na anyigbu m ma o bu akuda m.
12. A na m egwuri ufodu egwu di iche iche n’ime uche m ma o bu ata orji isi iji wepu uche m na ihe mgbu a.
13. di m ka ndu m di enweghi isi
14. A mara m na otu ubochi, otu onye ga agbatara m oso enye maaka mee ka ihe mgbu a puo nwa obere oge.
15. Ana m ekpere Chineke ka o ghara inote aka
16. Ana m ejisi ike ghara iche ya ka o bu ahu m, kama ka ihe iche na ebe m no.
17. A naghi m eche maka ihe mgbu a.
18. Ana m ejisie ike eche maka afo di n’ihu, etu ihe nile ga adi mgbe m chupugoro mgbu a.
19. A na m agwa onwe m na onaghi egbu m.
20. A na m agwa onwe m na agaghi m ekwe ka ihe mgbu a gbochie ihe o bula m kwesiri ime.
21. Anaghi m etinyere ya uche o bula.
22. E nwere m okwukwe na ebe ndi dibia bekee no, na otu ubochi aga enwe ogwugwo maka ihe mgbu m.
23. Na agbanyeghi ka osina di njo, a mara m na m ga anagide ya.
24. A na m eme ka aga asi na o noghi ebe ahu
25. na eche m echiche mgbe nile ma ihe a oga ebi
26. A na m eji obi m na atughari ihe obi uto mere mgbe gara aga.
27. A na m eche maka ndi mmadu o na ato m uto mu na ha imeko ihe
28. A na m ekpe ekpere ka ihe mgbu a kwusi.
29. A na m erube na obi m, ma o bu were ya na ihe mgbu a anoghi n’ime ahu m
30. A na m aga n’ihu eme ihe dika onweghi ihe mere
31. A na m ahuta ya dika ihe ima aka wee hapu ime ka o buru ihe ga enye m nsogbu ma o bu mekpaa m ahu.
32. Na agbanyeghi na o na egbu mgbu, a na m aga n’iru na eme ihe m na eme
33. na-adi m ka agaghi m anagidezi ya ozo
34. A na m agbali I na anoyere ndi mmadu ozo
35. A na m elaghara ya anya
36. A na m adabere na okwukwe m na Chineke.
37. na adim ka agaghi m emeli ihe a gaba n’iru
38. A na m eche maka ihe ndi na ato m uto ime.
39. A na m eme ihe o bula iji ewepu obi m na ihe mgbu a.
40. A na m eme ihe na eme m obi uto dika ile igwe onyonyo TV ma o bu ige egwu.
41. A na m eme ka agasi na o bughi akuku ahu m.
42. A na m eme ihe mmeghari ahu dika ije ozi ime ulo ma o bu oru ndi ozo.

**Dabere na ihe niile ị na-eme iji nagide mgbu gi, na otu ụbọchị, kedu njikwa ole o di gi ka i nwere ebe mgbu a no? Biko gwam ka m Kanye gburugburu (circle) na ihe ogugu nke kwesiri ma o bu Kanye ihe gburugburu na nke i choro. Na-echeta, ị nwere ike ikwu ma o bu Kanye na ihe ogugu o bula na ihe skelu a:**

0 1 2 3 4 5 6

Okweghi

njikwa Onweturu E nwere m

njikwa njikwa nke oma

**Dabere na ihe niile ị na-eme iji nagide mgbu gi, na otu ụbọchị, kedu etu ole i nwere ike ibelata ya? Biko gwam ka m Kanye gburugburu (circle) na ihe ogugu nke kwesiri ma o bu Kanye ihe gburugburu na nke i choro. Na-echeta, ị nwere ike ikwu ma o bu Kanye na ihe ogugu o bula na ihe skelu a:**

0 1 2 3 4 5 6

O kweghi m ebelata A na m ebelatatu ya A na m ebelata ya kpamkpam

ma oli
